# COVID-19: a new challenge for human beings

**DOI:** 10.1038/s41423-020-0407-x

**Published:** 2020-03-31

**Authors:** Penghui Yang, Xiliang Wang

**Affiliations:** 1 The Fifth Medical Center of Chinese PLA General Hospital, Beijing, China; 2 State Key Laboratory of Pathogen and Biosecurity, Beijing Institute of Microbiology and Epidemiology, Beijing, China

**Keywords:** Viral infection, Infection

Since December 2019, just a month before the Chinese Spring Festival, multiple cases of pneumonia of unknown etiology appeared in Wuhan, Hubei Province, China. Later, a novel coronavirus was identified in a bronchoalveolar lavage fluid sample from the Wuhan Seafood Market using metagenomic next-generation sequencing technology.^1^ On February 11, 2020, the virus was named severe acute respiratory syndrome coronavirus 2 (SARS-CoV-2) by the International Committee on Taxonomy of Viruses (ICTV). SARS-CoV-2 is the seventh member of the coronavirus family that can infect humans after the emergence of severe acute respiratory syndrome coronavirus (SARS-CoV) and Middle East respiratory syndrome coronavirus (MERS-CoV). The World Health Organization announced that the novel coronavirus pneumonia epidemic caused by SARS-CoV-2 was classified as a public health emergency of international concern on January 30, 2020. The new coronavirus disease caused by SARS-CoV-2 was named coronavirus disease 2019 (COVID-19). As of March 9, 2020, a total of 80,905 laboratory-confirmed COVID-19 cases with 3123 deaths have been reported in mainland China, and the epidemic has spread to >100 countries worldwide.^2^ China has incorporated COVID-19 into class B infectious diseases, as stipulated by the law of the People’s Republic of China for the prevention and control of infectious diseases, and has taken preventive and control measures in accordance with Class A infectious diseases. The unprecedented number of COVID-19 cases not only in China but also in many countries has triggered the alarm for public health to respond to emerging and re-emerging diseases. A comprehensive strategy, including surveillance, diagnostics, clinical treatment, research, and development of vaccines and drugs, is urgently needed to win the battle against COVID-19 and other infectious diseases.

## Etiology of SARS-CoV-2

Coronaviruses are not new infectious pathogens in the world. The first described coronavirus was isolated from chickens in 1937. Human coronaviruses were first identified in the mid-1960s (https://www.cdc.gov/coronavirus/index.html). Coronaviruses belong to the Coronaviridae family. Coronaviridae is a family of enveloped, single-stranded, positive-sense RNA virus. The total length of the genome is 30 Kb, consisting of a 5’-terminal noncoding region, an open reading box (ORF) 1a/b-coding region, an s region encoding the spike glycoprotein (S protein), an e region encoding the envelope protein (E protein), an m region encoding the membrane protein (M protein), an n region encoding the nucleocapsid protein (N protein), and a -3’-terminal noncoding region. Among them, the poly protein encoded in the ORF1a/b region of the nonstructural protein can be cut by 3CLpro and PLpro of the virus to form RNA-dependent RNA polymerase and helicase, which can guide the replication, transcription, and translation of the virus genome.^3^ The structural protein S can specifically bind to the receptor of the host cell, and this is the key protein for viruses to invade susceptible cells. The M and E proteins are involved in the formation of the virus envelope, while the N protein is involved in the assembly of the virus.

According to the genome structure and phylogenetic analysis of coronaviruses, the Coronaviridae family can be divided into four genera: α, β, γ, and δ. The coronaviruses of the α and β genera generally infect mammals and humans, while the coronaviruses of the γ and δ genera mainly infect birds. SARS-CoV-2 is a novel coronavirus of the β genus; it is round or oval, with a diameter of approximately 60–140 nm and a crown-shaped appearance under an electron microscopy.^4^ Besides SARS-CoV-2, six other coronaviruses can infect humans, including human coronavirus 229E (HCoV-229E), OC43 (HCoV-OC43), NL63 (HCoV-NL63), HKU1 (HCoV-HKU1), SARS-CoV, and MERS-CoV. A protein sequence analysis showed that the amino acid similarity of the seven conserved nonstructural proteins between SARS-CoV-2 and SARS-CoV was 94.6%, suggesting that they might belong to the same species. The homology between the SARS-CoV-2 genome and the bat SARS-like coronavirus (Bat-CoV (RaTG13)) genome is 96%.^5^

Coronaviruses are sensitive to heat and ultraviolet rays. They can be stored for several years at −80 °C and inactivated at 56 °C for 30 min (the most commonly used method to inactivate SARS-CoV-2 in the laboratory). In addition, 75% ethanol, peracetic acid, and chlorine-containing disinfectants can effectively inactivate SARS-CoV-2.^5^

## Pathogenicity of SARS-CoV-2

Coronavirus can cause human respiratory tract infection or animal intestinal infection. The process of virus infection requires the participation of receptors on the surface of the host cell membrane. The S protein on the surface of coronavirus can recognize and bind to the receptor and then invade the host cell through clathrin-mediated endocytosis.^6^ Different coronaviruses can use different cell receptors to complete the invasion. For example, the receptor of HCoV-229E is aminopeptidase N (also known as CD13), the receptor of SARS-CoV is ACE2,^7^ and the receptor of MERS-CoV is DPP4 (also known as CD26).^8^ It has been indicated that aminopeptidase N and DPP4 are not receptors of SARS-CoV-2 and that ACE2 can be used as its receptor.^5^ The pathogenicity of HCoV-229E, HCoV-OC43, HCoV-NL63, and HCoV-HKU1 is relatively low, generally only causing slight respiratory symptoms. SARS-CoV and MERS-CoV can cause SARS and MERS, respectively. Both caused outbreaks with severe symptoms and a high mortality.^7,8^ Pathological findings of COVID-19 revealed that the overactivation of T cells, manifested by an increase in Th17 and the high cytotoxicity of CD8 T cells, partially accounts for the severe immune injury.^9^ People are generally susceptible to SARS-CoV-2, with an incubation period of 1–14 days, with an average of 3–7 days; the main source of infection is COVID-19 patients, and asymptomatic patients may also be the source of infection.^10,11^ The main route of transmission is respiratory droplets, and people can also be infected by coming into contact with articles contaminated with virus droplets. In addition, a study showed that SARS-CoV-2 nucleic acid can be detected in the feces and urine of patients with COVID-19, suggesting that SARS-CoV-2 may be transmitted through the digestive tract through the fecal–oral route. The main symptoms of COVID-19 caused by SARS-CoV-2 are fever, dry cough, and fatigue. A few patients may have runny nose, sore throat, and diarrhea. Some patients may have dyspnea, and those who have a serious form of COVID-19 may rapidly progress to acute respiratory distress syndrome, coagulation dysfunction, and septic shock.^11,12^ From the existing treatment cases, mild patients only show low fever and slight fatigue but no pneumonia. Most patients have a good prognosis, a few patients may have a severe condition, and the elderly and those with chronic basic diseases have a poor prognosis.

## COVID-19 detection and diagnosis

With the successful virus isolation and genome sequencing of SARS-CoV-2, the current diagnosis mainly depends on quantitative reverse transcriptase polymerase chain reaction to detect the SARS-CoV-2 nucleic acid. The novel coronavirus is highly homologous to the known SARS-CoV-2 coronavirus in respiratory specimens or blood samples and can be used as a diagnostic standard for SARS-CoV-2 infection.^13^ Recently, immunoglobulin M (IgM) and IgG antibody detection reagents and SARS-CoV-2 antigen detection reagents established by colloidal gold and enzyme-linked immunosorbent technologies have also been successfully developed and applied for auxiliary diagnosis.

## COVID-19 prevention and control

The basic principles of prevention and control of infectious diseases are to eliminate the source of infection, cut off the route of transmission, and protect the susceptible population. SARS-CoV-2 is mainly transmitted through respiratory droplets and contact. Necessary personal protective measures are helpful to control the spread of SARS-CoV-2. A vaccine is an effective measure to protect susceptible populations. To date, there are no SARS-CoV-2 vaccines available or clinical trials for vaccinations, but several domestic and foreign research institutions and enterprises have used several methods, including mRNA nanovaccine technology, recombinant or inactivated vaccine technology, and DNA vaccine technology, to develop a SARS-CoV-2 vaccine. For example, Moderna, a biotech company, is working with the National Institutes of Health on a potential mRNA vaccine candidate against COVID-19. AbMax Biotechnology Co., Ltd. announced that they have successfully developed a SARS-CoV-2 antibody.

## COVID-19 clinical treatment

### Symptomatic treatment and supportive therapy

Currently, symptomatic treatment and supportive therapy are mainly adopted for patients with COVID-19, and these include the treatment of basic diseases, symptom relief, effective protection and supportive treatment of internal organs, active prevention and treatment of complications, and respiratory support if necessary. More attention should be paid for maintaining the balance of water and electrolytes and maintaining the stability of the internal environment. Glucocorticoids can be used for a short time according to the degree of dyspnea and the progress of chest imaging.

### Antiviral therapy

Currently, there are no specific anti-SARS-CoV-2 drugs in the clinic. The most efficient research strategy is “old drug, new use.” Remdesivir (GS-5734), a drug under development by Gilead Sciences in the United States, is a nucleoside analog prodrug that can inhibit Ebola virus, thereby playing an antiviral role.^14^ In vitro and in vivo experiments confirmed that a low dose of remdesivir can inhibit SARS-CoV and MERS-CoV. It has a good inhibitory effect. Remdesivir, with complete pharmacokinetic results and good safety, may be one of the most promising drugs against SARS-CoV-2 pneumonia. Currently, under the leadership of China Japan Friendship Hospital, a phase III clinical trial of radcivir for the treatment of COVID-19 has been officially launched in Wuhan Jinyintan Hospital on February 5, 2020, and its efficacy will be determined using a strict clinical double-blind test validation. Recent studies have shown that radcivir and chloroquine exhibit good inhibitory effects on SARS-CoV-2 in vitro.^15^ In addition, according to the novel coronavirus pneumonia diagnosis and treatment plan (trial version sixth), inhalation of alpha interferon inhalation and ralproveravir/ritonavir or intravenous injection of ribavirin can also be administered.^13^

### Chinese medicine treatment

COVID-19 belongs to the category of Chinese medicine for epidemic diseases. The cause of the disease is viral infection. Different people can be treated based on the syndrome differentiation: the early stage (cold dampness and stagnant lung), the middle stage (pestilence and toxin closing lung), the severe stage (internal closure and external removal), and the recovery stage (deficiency of lung, spleen, and qi). To date, at least 54 preventive, observational, and interventional drug studies have been registered in the national clinical trial registration center, involving a variety of traditional Chinese and Western medicines, and these include Lianhua Qingwen capsule, chloroquine, darunavir/corbistar, etc.

SARS-CoV-2 is now turning into a major challenge in China. The sudden outbreak of COVID-19 once again proves that biosafety is an indispensable part of human security. Bats are only the natural hosts of SARS-CoV-2. There should be one or two intermediate hosts of wild animals between the natural host and humans. If wild animals are not treated well, humans may be punished by nature. Currently, the number of cases of COVID-19 infection is still increasing. To further eliminate the source of infection, prevent the route of transmission, protect the susceptible population, and achieve early detection, early isolation and treatment must depend on the joint efforts of clinical and medical treatment, public health, and basic research. To fight this disease, we need to be well prepared at the frontline to stay ahead of this epidemic.
